# Supplementary material for: Heavily and fully modified RNAs guide efficient SpyCas9-mediated genome editing
Source: Nat Commun. 2018 Jul 6;9:2641. doi: 10.1038/s41467-018-05073-z (PMC6035171; doi:10.1038/s41467-018-05073-z)
Supplement: Supplementary file 1 — Supplementary Information [file 41467_2018_5073_MOESM1_ESM.pdf]

SUPPORTING INFORMATION

Heavily and Fully Modified RNAs Guide Efficient SpyCas9-Mediated Genome Editing

Aamir Mir[†], Julia F. Alterman[†], Matthew R. Hassler[†], Alexandre J. Debacker[†], Edward Hudgens [§], Dimas Echeverria[†], Michael Brodsky[§], Anastasia Khvorova[*†‡], Jonathan K. Watts[*†‖], and Erik J. Sontheimer[*†‡]

[†]RNA Therapeutics Institute
[§]Department of Molecular, Cell and Cancer Biology
[‡]Program in Molecular Medicine
[‖]Department of Biochemistry and Molecular Pharmacology
University of Massachusetts Medical School
Worcester, Massachusetts 01605
United States

Supplementary Figure 1	The gating strategy used for flow cytometry analysis of HEK293T-TLR cells after electroporation
Supplementary Figure 2	Lipid transfections of HEK293T-TLR cells with **C0**, **C1**, **C2** and **C3**
Supplementary Figure 3	TIDE editing efficiencies of Cas9 loaded with different modified RNAs in HEK293T-TLR cells
Supplementary Figure 4	*In vitro* DNA cleavage assays of modified crRNAs and tracrRNAs
Supplementary Figure 5	Comparison of synthetic crRNAs and tracrRNAs using 3 picomoles of Cas9 RNP
Supplementary Figure 6	Serum stabilities of crRNAs **C21**, **C0**, and **C20** and tracrRNAs **T0**, **T2**, and **T8**
Supplementary Figure 7	Testing of crRNA designs **C10, C20** and **C21** tested with tracrRNAs **T2** and **T8** using 3 pmoles of Cas9-RNP in HEK293T cells
Supplementary Figure 8	Effects of modified RNAs on on-target vs. off-target editing for target sites *VEGFA* and *HBB*
Supplementary Figure 9	Comparison of sequence-optimized crRNA and tracrRNA series in HEK293T-TLR cells
Supplementary Table 1	Sequences and modification patterns of all the crRNAs and tracrRNAs synthesized for this study
Supplementary Table 2	Target sites and primers used for TIDE analysis in this study
Supplementary Table 3	Percent purity of compounds synthesized in this study
Supplementary Data 1	Raw data for genome editing experiments in HEK293T-TLR cells and hESCs

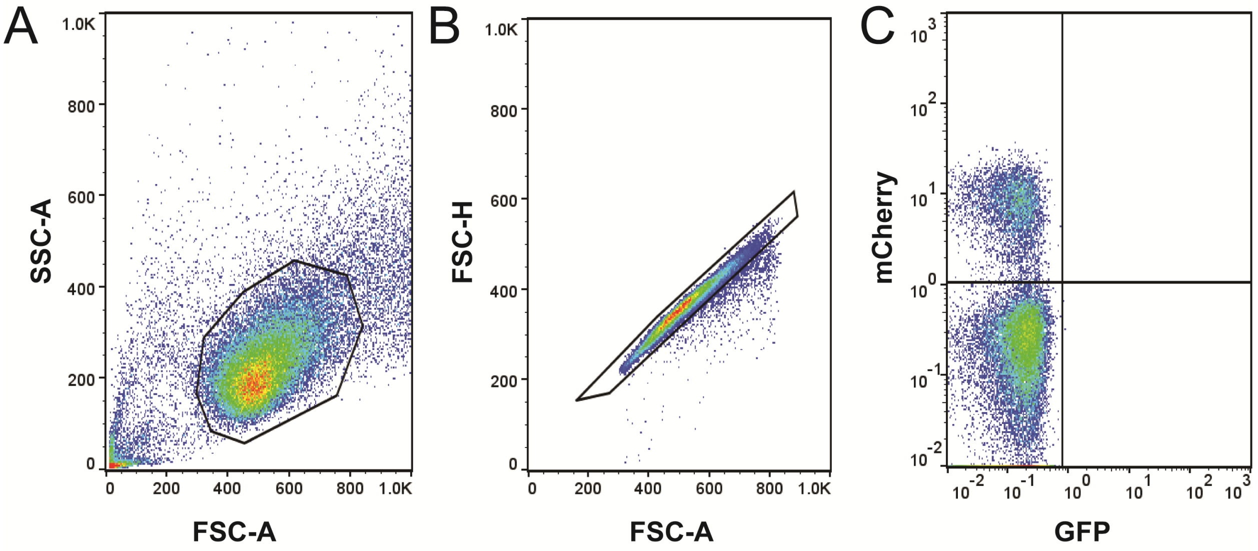

Supplementary Figure 1: Flow cytometry analysis of HEK293T-TLR cells after electroporation. The HEK293T cells were first gated based on forward and side scattering to remove cell debris (**a**), then gated to select single cells (**b**), and then finally gated to select mCherry-positive cells (**c**). The mCherry-positive cells are in the top left quadrant in **c**. The quadrant gate was based on the “no sgRNA” control in every experiment.

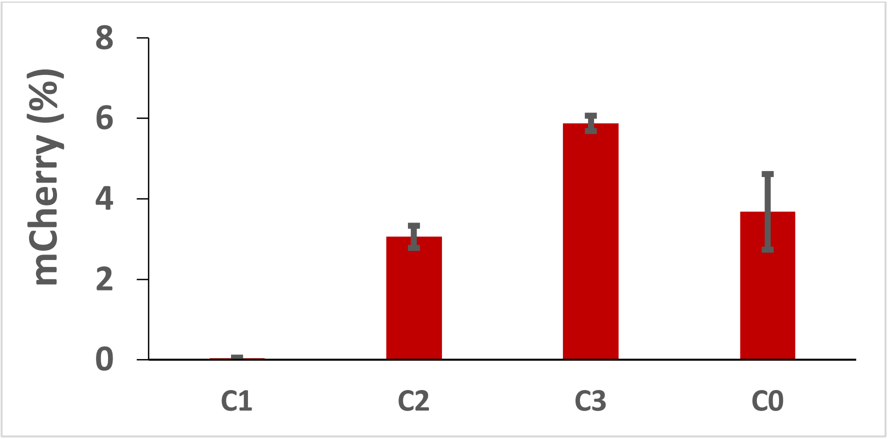

Supplementary Figure 2: HEK293T-TLR cells were transfected with Lipofectamine CRISPRMAX Cas9 Transfection Reagent and analyzed by flow cytometry for mCherry-positive cells. Error bars show ± SD of three biological replicates.

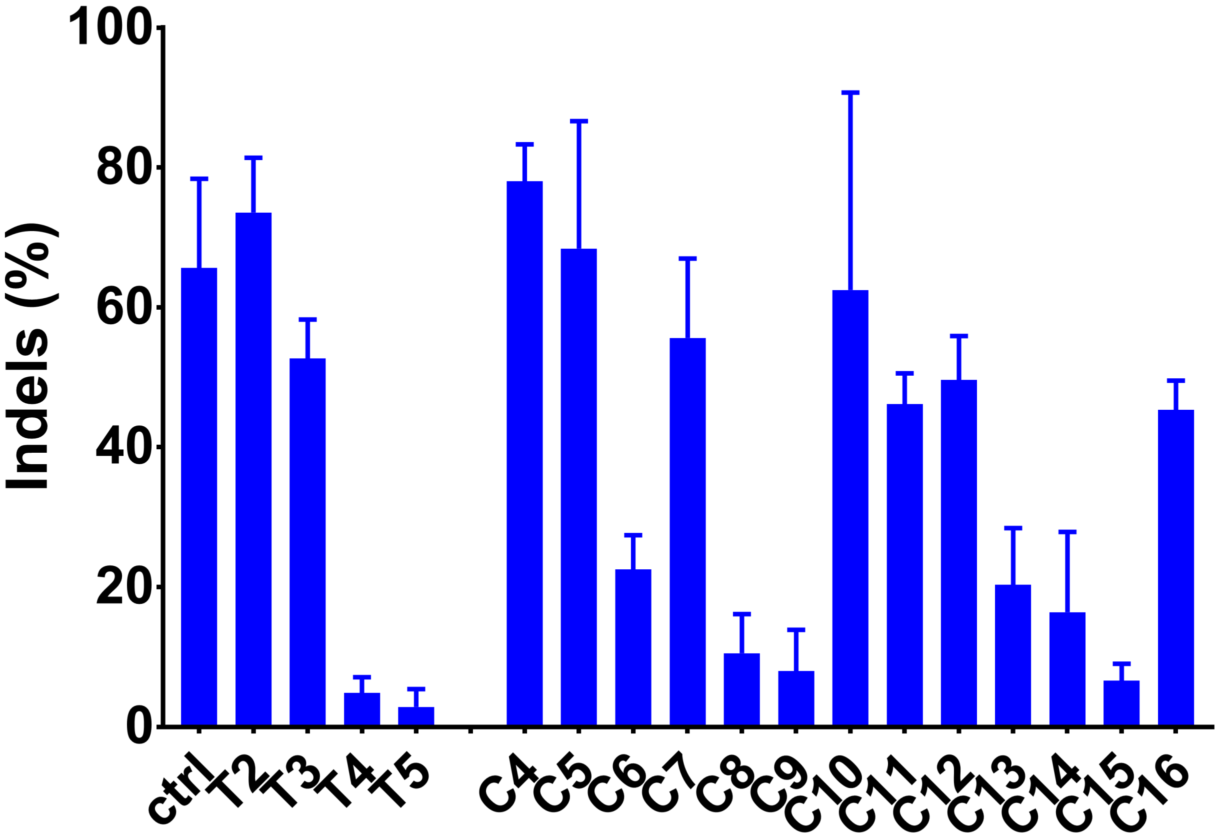

Supplementary Figure 3: Overall editing efficiencies of Cas9 loaded with different modified RNAs. The chemically modified crRNAs **C4-C16** and tracrRNAs **T2-T5** were electroporated into HEK293T-TLR cells and the resulting indel rate was determined using TIDE analysis. The modified crRNAs and tracrRNAs were tested against IDT purchased corresponding RNAs. The ctrl refers to IDT purchased crRNA:tracrRNA pair. Error bars represent standard deviations resulting from 3-5 biological replicates.

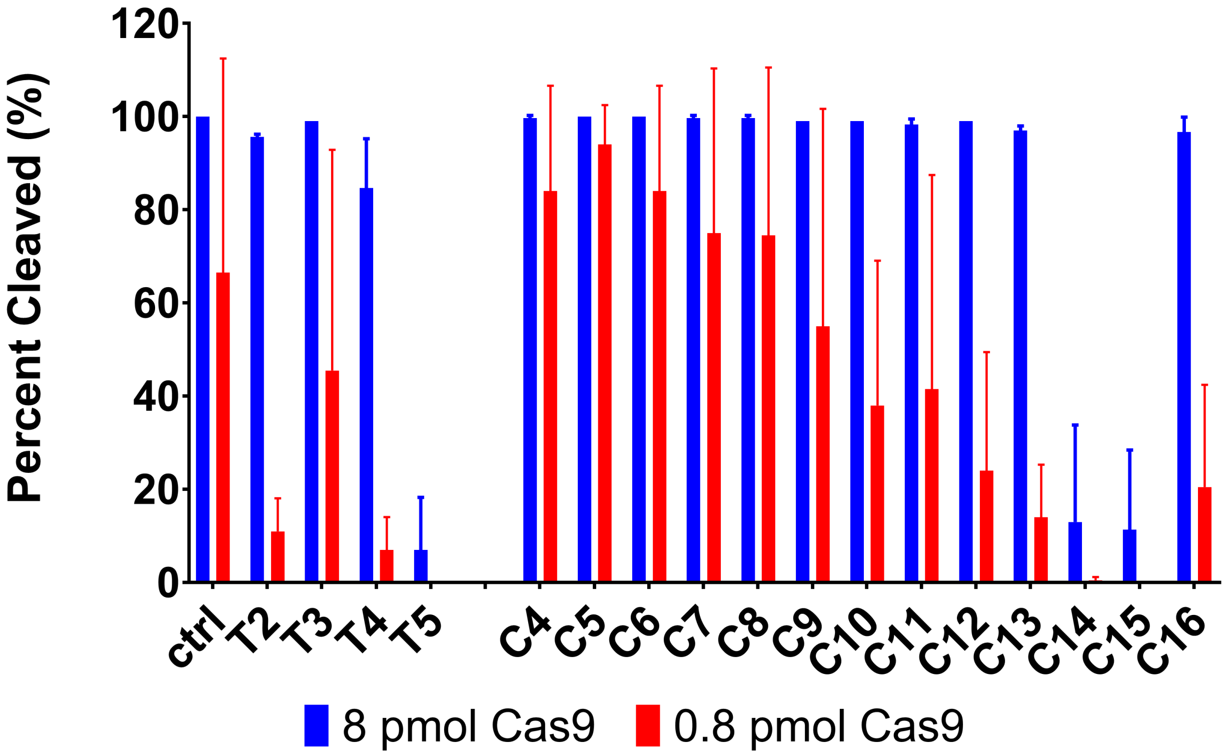

Supplementary Figure 4: *In vitro* DNA cleavage assays to determine the functionality of modified RNAs. DNA cleavage assays were performed using saturating (8 pmols) and sub-saturating (0.8 pmols) amounts of Cas9 RNP complex. The modified crRNAs and tracrRNAs were tested against IDT purchased corresponding RNAs. The ctrl refers to IDT purchased crRNA:tracrRNA pair. Error bars represent standard deviations resulting from at least two replicates.

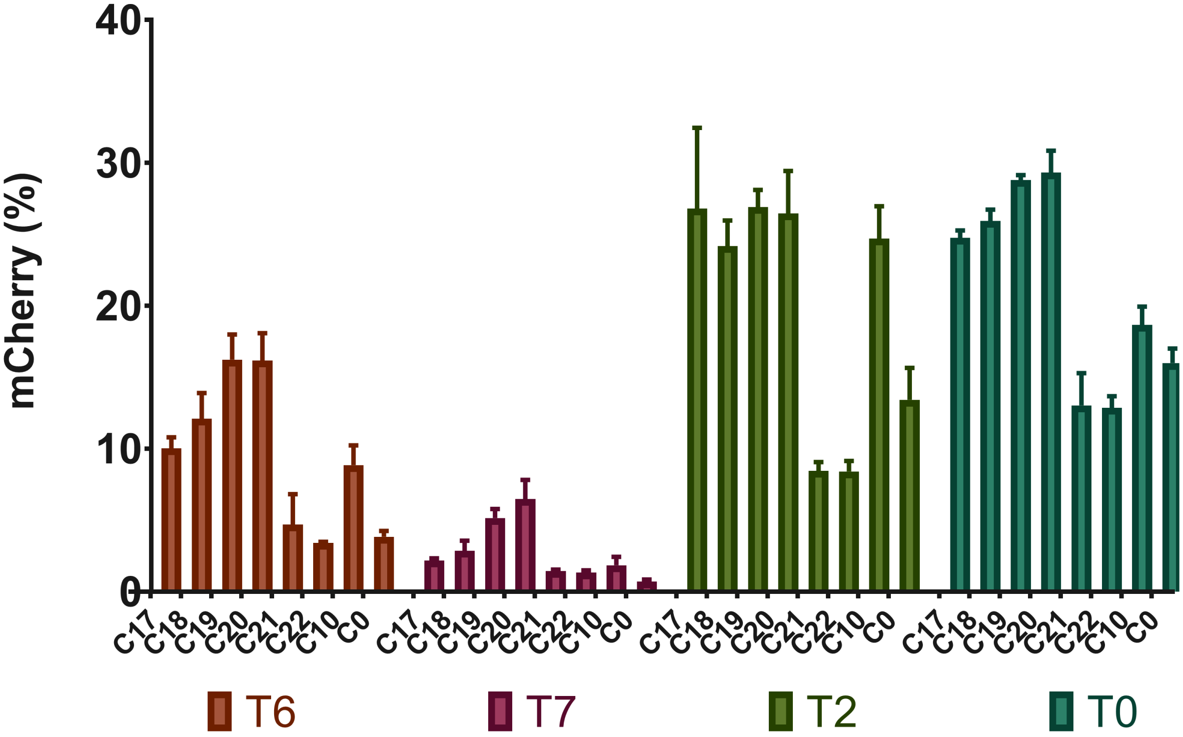

Supplementary Figure 5: Comparison of synthetic crRNAs and tracrRNAs using 3 picomoles of Cas9 RNP (compare to Fig. 3 in the main text). Error bars show ± SD of three biological replicates.

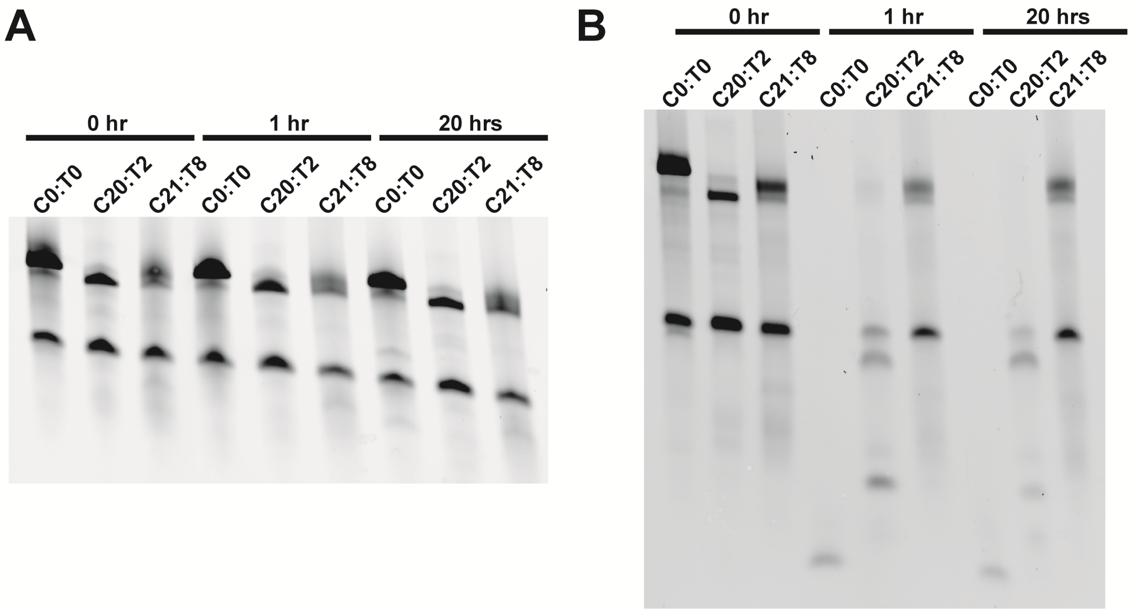

Supplementary Figure 6: Serum stability of crRNAs C21, C0, C20 and tracrRNAs T0, T2, T8. The indicated crRNA:tracrRNA combinations were used to make Cas9 RNP complex that was then incubated with cleavage buffer (**a**) or 8% FBS (**b**) for 0, 1 and 20 hrs. The reactions were then treated with Proteinase K and then resolved on a 10% denaturing polyacrylamide gel. The gels were stained with SYBR Safe dye and then imaged on Typhoon FLA imager. The upper band in all lanes corresponds to tracrRNA and lower band corresponds to crRNA.

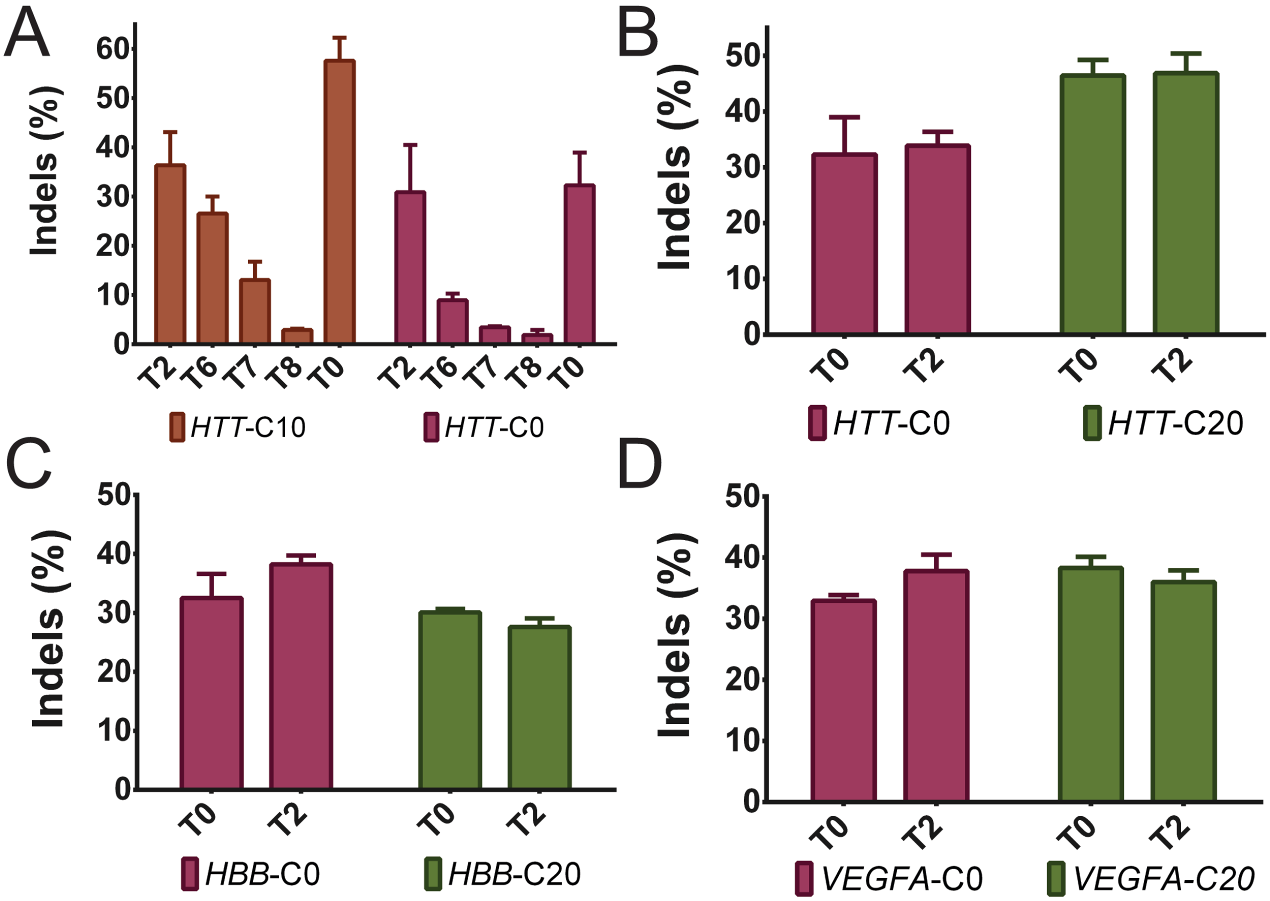

Supplementary Figure 7. The crRNA designs **C10, C20** and **C21** tested with the indicated tracrRNAs using 3 pmoles of Cas9-RNP in HEK293T cells (compare to Fig. 4 in the main text). Mean values from triplicate experiments (± SD) are shown. Three endogenous target sites were tested, namely *HTT* (**a**,**b**), *HBB* (**c**) and *VEGFA* (**d**). Error bars show ± SD of three biological replicates.

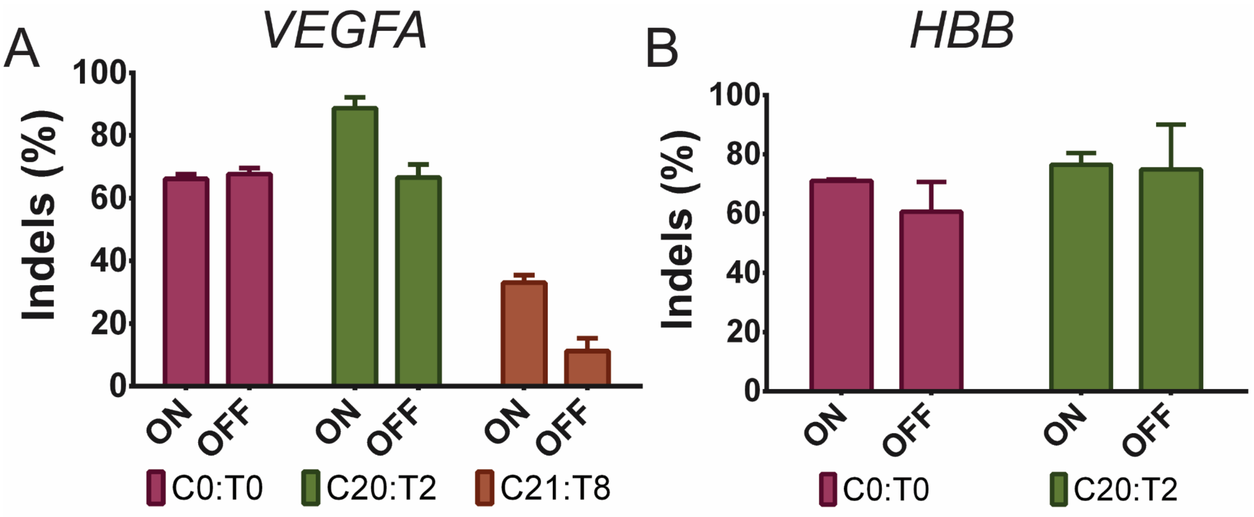

Supplementary Figure 8. Effects of modified RNAs on on-target vs. off-target editing for target sites *VEGFA* (**a**) and *HBB* (**b**). Error bars show ± SD of at least three biological replicates.

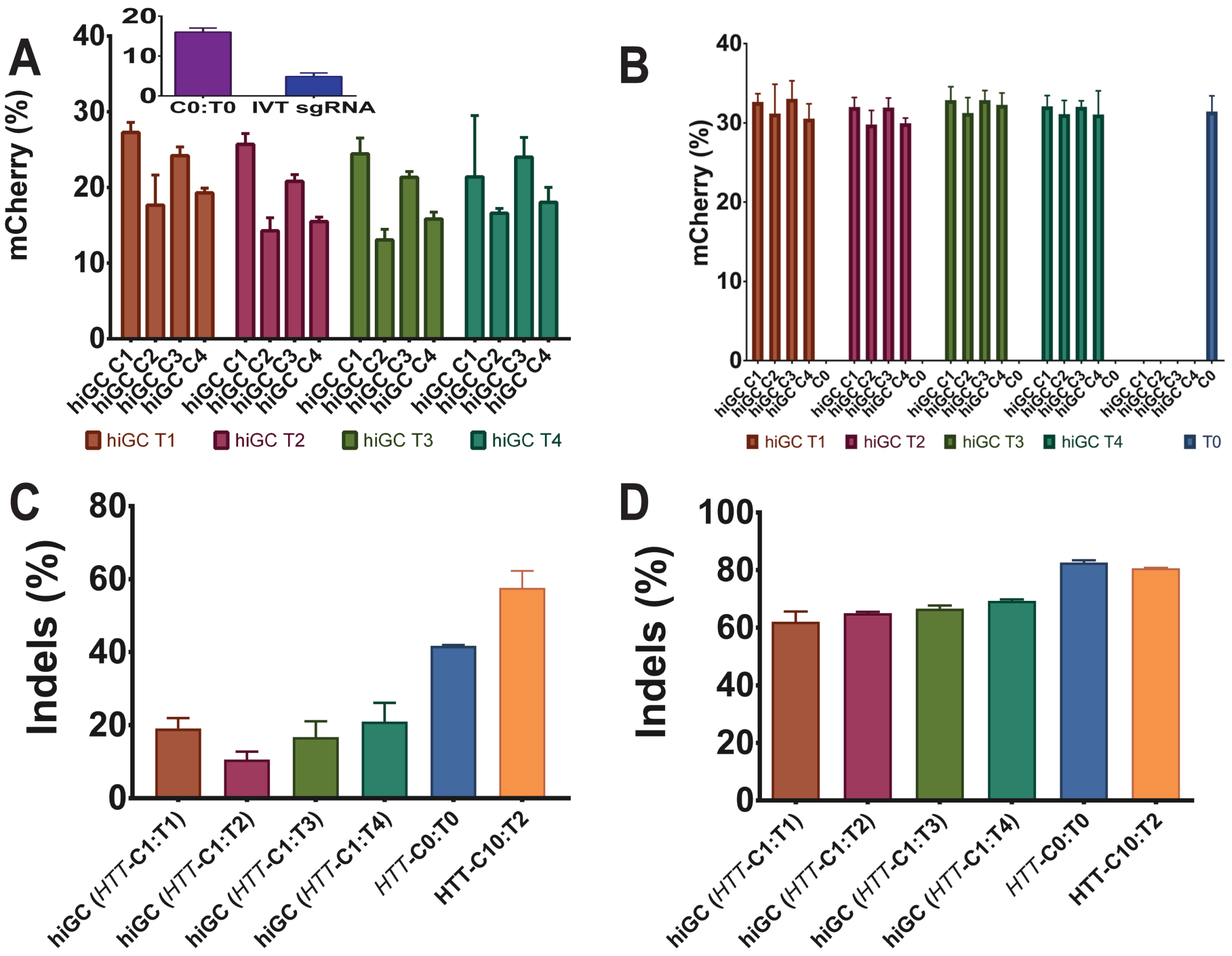

Supplementary Figure 9: Comparison of sequence-optimized crRNA and tracrRNA series. HEK293T cells were electroporated with 3 pmoles (**a**,**c**) or 20 pmoles (**b**,**d**) of Cas9 RNP. Two endogenous sites were targeted in HEK293T cells: the TLR site (**a**,**b**) and the genomic *HTT* site (**c**,**d**). The indel efficiency was determined using TIDE, whereas the fractions of mCherry-positive cells were measured using flow cytometry. *In vitro* synthesized (IVT) sgRNA was also included as a control in **a**. Error bars represent ±SD from 3 biological replicates.

Supplementary Table 1: All the crRNAs and tracrRNAs synthesized for this study.

KEY: N = RNA, N = 2'-O-methyl RNA, N = 2'-fluoro RNA
N = 3' phosphorothioate

Name	Sequence	Extinction Coefficient	MW (Observed/ Calculated)
**C1**	GGUGAGCUCUUAUUUGCGUAGUUUUAGAGCUAUGCU	352710	11942.8/ 11942.8
**C2**	GGUGAGCUCUUAUUUGCGUAGUUUUAGAGCUAUGCU	352710	11739.7/ 11740.3
**C3**	GGUGAGCUCUUAUUUGCGUAGUUUUAGAGCUAUGCU	352710	11788.6/ 11788.5
**C4**	GGUGAGCUCUUAUUUGCGUAGUUUUAGAGCUAUGCU	352710	11888.6/ 11888.7
**C5**	GGUGAGCUCUUAUUUGCGUAGUUUUAGAGCUAUGCU	352710	11832.6/ 11832.6
**C6**	GGUGAGCUCUUAUUUGCGUAGUUUUAGAGCUAUGCU	352710	11832.6/ 11832.6
**C7**	GGUGAGCUCUUAUUUGCGUAGUUUUAGAGCUAUGCU	352710	11916.7/ 11916.8
**C8**	GGUGAGCUCUUAUUUGCGUAGUUUUAGAGCUAUGCU	352710	11916.6/ 11916.8
**C9**	GGUGAGCUCUUAUUUGCGUAGUUUUAGAGCUAUGCU	352710	12012.5/ 12013.2
**C10**	GGUGAGCUCUUAUUUGCGUAGUUUUAGAGCUAUGCU	352710	12024.5/ 12025.1
**C11**	GGUGAGCUCUUAUUUGCGUAGUUUUAGAGCUAUGCU	352710	12052.4/ 12053.3
**C12**	Cy3-GGUGAGCUCUUAUUUGCGUAGUUUUAGAGCUAUGCU	357610	12654.8/ 12655.5
**C13**	Cy3-GGUGAGCUCUUAUUUGCGUAGUUUUAGAGCUAUGCU	357610	12682.8/ 12683.6
**C14**	Cy3-GGUGAGCUCUUAUUUGCGUAGUUUUAGAGCUAUGCUAAA-TegChol	399190	14488.5/ 14489.3
**C15**	Cy3-GGUGAGCUCUUAUUUGCGUAGUUUUAGAGCUAUGCUAAA-TegChol	399190	14516.4/ 14517.5
**C16**	Cy3-GGUGAGCUCUUAUUUGCGUAGUUUUAGAGCUAUGCUAAA-GalNAc	399190	15521.2/ 15520.2
**C17**	GGUGAGCUCUUAUUUGCGUAGUUUUAGAGCUAUGCU	352710	12027.4/ 12027.2
**C18**	GGUGAGCUCUUAUUUGCGUAGUUUUAGAGCUAUGCU	352710	12026.4/ 12027.2
**C19**	GGUGAGCUCUUAUUUGCGUAGUUUUAGAGCUAUGCU	352710	12029.4/ 12029.2
**C20**	GGUGAGCUCUUAUUUGCGUAGUUUUAGAGCUAUGCU	352710	12000.4/ 12001.1
**C21**	GGUGAGCUCUUAUUUGCGUAGUUUUAGAGCUAUGCU	352710	11916.6/ 11916.6
**C22**	GGUGAGCUCUUAUUUGCGUAGUUUUAGAGCUAUGCU	352710	11972.5/ 11972.9
**hiGC C1**	GGUGAGCUCUUAUUUGCGUAGUUUUAGAGCGAGCGC	353160	12077.5/ 12078.1
**hiGC C2**	GGUGAGCUCUUAUUUGCGUAGUUUUAGAGCGAGCGC	353160	12005.4/ 12005.9
**hiGC C3**	GGUGAGCUCUUAUUUGCGUAGUUUUAGAGCGAGCGC	353160	12041.4/ 12042
**hiGC C4**	GGUGAGCUCUUAUUUGCGUAGUUUUAGAGCGAGCGC	353160	12029.4/ 12030
**T1**	AGCAUAGCAAGUUAAAAUAAGGCUAGUCCGUUAUCAACUUGAAAAAGUGGCACCGAGUCGGUGCUUU	699750	22130.8/ 22130.2
**T2**	AGCAUAGCAAGUUAAAAUAAGGCUAGUCCGUUAUCAACUUGAAAAAGUGGCACCGAGUCGGUGCUUU	699750	22439.6/ 22438.8
**T3**	AGCAUAGCAAGUUAAAAUAAGGCUAGUCCGUUAUCAACUUGAAAAAGUGGCACCGAGUCGGUGCUUU	699750	22632.5/ 22631.6
**T4**	AGCAUAGCAAGUUAAAAUAAGGCUAGUCCGUUAUCAACUUGAAAAAGUGGCACCGAGUCGGUGCUUU	699750	22453.6/ 22452.8
**T5**	AGCAUAGCAAGUUAAAAUAAGGCUAGUCCGUUAUCAACUUGAAAAAGUGGCACCGAGUCGGUGCUUUAAA-TegChol	741330	24287.4/ 24286.7
**T6**	AGCAUAGCAAGUUAAAAUAAGGCUAGUCCGUUAUCAACUUGAAAAAGUGGCACCGAGUCGGUGCUUU	699750	22449.2/ 22448.7
**T7**	AGCAUAGCAAGUUAAAAUAAGGCUAGUCCGUUAUCAACUUGAAAAAGUGGCACCGAGUCGGUGCUUU	699750	22453.1/ 22452.7
**T8**	AGCAUAGCAAGUUAAAAUAAGGCUAGUCCGUUAUCAACUUGAAAAAGUGGCACCGAGUCGGUGCUUU	699750	22463.2/ 22462.7
**hiGC T1**	GCGCUCGCAAGUUAAAAUAAGGCUAGUCCGUUAUCAACUUGAAAAAGUGGCACCGAGUCGGUGCUUU	681480	22407.4/ 22406.7
**hiGC T2**	GCGCUCGCAAGUUAAAAUAAGGCUAGUCCGUUAUCAACUUGAAAAAGUGGCACCGAGUCGGUGCUUU	681480	22335/ 22334.5
**hiGC T3**	GCGCUCGCAAGUUAAAAUAAGGCUAGUCCGUUAUCAACUUGAAAAAGUGGCACCGAGUCGGUGCUUU	681480	22371.3/ 22370.6
**hiGC T4**	GCGCUCGCAAGUUAAAAUAAGGCUAGUCCGUUAUCAACUUGAAAAAGUGGCACCGAGUCGGUGCUUU	681480	22335/ 22334.5
***HTT*-C10**	UGAAGUGCACACAGUAGAUGGUUUUAGAGCUAUGCU	372510	12092.5/12093.2
***HTT*-C20**	UGAAGUGCACACAGUAGAUGGUUUUAGAGCUAUGCU	363200	12092.5/ 12093.2
***HTT*-C21**	UGAAGUGCACACAGUAGAUGGUUUUAGAGCUAUGCU	359820	12090.6/12090.7
***HTT*-hiGC 1**	UGAAGUGCACACAGUAGAUGGUUUUAGAGCGAGCGC	372960	12121.5/12122.2
***HTT* C0**	UGAAGUGCACACAGUAGAUGGUUUUAGAGCUAUGCU	372510	11679.3/11672.3

**C0**	GGUGAGCUCUUAUUUGCGUAGUUUUAGAGCUAUGCU	352710	11563.3/11564.1
**T0**	Edit-R tracrRNA (Dharmacon)	--	--
***HBB*-C0**	CUUGCCCCACAGGGCAGUAAGUUUUAGAGCUAUGCU	351450	11583.2
***HBB*-C20**	CUUGCCCCACAGGGCAGUAAGUUUUAGAGCUAUGCU	363200	12003.5/ 12004.1
**VEGFA-C0**	GGUGAGUGAGUGUGUGCGUGGUUUUAGAGCUAUGCU	IDT	purchased
**VEGFA-C20**	GGUGAGUGAGUGUGUGCGUGGUUUUAGAGCUAUGCU	363200	12175.5/ 12175.1
**VEGFA-C21**	GGUGAGUGAGUGUGUGCGUGGUUUUAGAGCUAUGCU	372510	12008.6/ 12008.7

Supplementary Table 2: Target sites and primers used for TIDE analysis in this study.

Target Site	Sequence (5'-3')	Location (GRCh38)	Forward Primer (Sequencing Primer)	Reverse Primer
HTT ON	TGAAGTGCACACAGT AGATGAGG	Chr4:3214123-3214101	TGTCAGAGCTGTCCTTCTGG	ACAAAGCTTCAAAACGCCCT
HBB ON	CTTGCCCCACAGGG CAGTAACGG	Chr11:5 226968-5 226990	ACGGCTGTCATCACTTAGAC	CCCTGTTACTTATCCCCTTCC
HBB OFF	TCAGCCCCACAGGG CAGTAAGGG	Chr9:101833584-101833606	CTGAGGAGGAAACACATAATGAG	AAGCCATTAGCTAGGTGTCGA
VEGFA ON	GGTGAGTGAGTGTG TGCGTGTGG	Chr6:43769717-43769739	GTAGCTGTTTGGGAGGTCAG	TCTGCGGACGCTCAGTGAA
VEGFA OFF	TGTGGGTGAGTGTG TGCGTGAGG	Chr5: 116098962-116098984	AGCCCTCGCTAGATACTGA	GGATGAACCTGGAGGGTGT

Supplementary Table 3: Percent purity of compounds synthesized in this study.

Compound ID	Mass Calc.	Mass Found	% Purity RP-UV[a]	% Purity by Mass[b]
**C1**	11942.8	11943.3	86.7	68.8
**C2**	11740.3	11740.8	89.9	69.2
**C3**	11788.5	11789.1	87.9	66.2
**C4**	11888.7	11889.2	88.3	74.0
**C5**	11832.6	11833.2	95.3	78.8
**C6**	11832.6	11833.2	96.5	79.5
**C7**	11916.8	11917.3	82.4	78.3
**C8**	11916.8	11917.2	93.6	78.4
**C9**	12013.2	12013.6	50.9	61.3
**C10**	12025.1	12025.5	62.9	63.2
**C11**	12053.3	12053.7	60.4	53.2
**C12**	12655.5	12655.5	90.3	61.3
**C13**	12683.6	12683.6	90.1	61.8
**C14**	14489.3	14489.3	87.0	50.2
**C15**	14517.5	14517.3	84.2	40.7
**C16**	15520.2	15523.2	91.6	54.5
**C17**	12027.2	12027.6	68.2	61.4
**C18**	12027.2	12027.6	71.7	63.1
**C19**	12029.2	12029.6	72.2	61.1
**C20**	12001.1	12001.6	67.0	57.9
**C21**	11916.6	11917.2	51.8	52.0
**C22**	11972.9	11973.5	65.8	56.4
**hiGC C1**	12078.1	12078.6	67.0	61.1
**hiGC C2**	12005.9	12006.4	57.9	57.3
**hiGC C3**	12042.0	12042.5	63.6	60.2
**hiGC C4**	12030.0	12030.5	62.8	61.2
**T1**	22130.2	22130.6	79.3	51.8
**T2**	22438.8	22439.3	80.4	55.4
**T3**	22631.6	22632.2	76.3	52.8
**T4**	22452.8	22453.3	77.2	58.5
**T5**	24286.7	24287.2	83.1	37.8
**T6**	22448.7	22449.2	74.0	47.9
**T7**	22452.7	22453.1	79.7	45.4
**T8**	22462.7	22463.2	69.9	43.8
**hiGC T1**	22406.7	22407.3	72.0	51.4
**hiGC T2**	22334.5	22335.0	71.1	46.0
**hiGC T3**	22370.6	22371.1	67.9	49.4
**hiGC T4**	22334.5	22335.1	78.4	49.5
**HTT-C10**	12093.2	12093.7	84.1	70.5

**HTT-C20**	12093.2	12093.7	84.4	66.9
**HTT-C21**	12090.7	12091.4	74.5	64.0
**HTT-hiGC1**	12122.2	12122.6	72.9	61.0
**HTT-C0**	11672.3	11679.8	90.5	66.3
**C0**	11564.1	11564.5	65.0	50.2
**T0**	Dharmacon	Dharmacon	Dharmacon	Dharmacon
**HBB-C0**	11583.2	11584.0	55.4	57.7
**HBB-C20**	12004.1	12004.6	93.3	66.2
**VEGFA-C0**	IDT	IDT	IDT	IDT
**VEGFA-C20**	12175.1	12175.6	70.6	53.5
**VEGFA-C21**	12008.7	12009.2	85.5	52.2

[a] We estimate this % purity by integrating the UV absorbance LC trace from LCMS; the percentage represents the area of the desired compound peak (excluding shoulders) relative to the area of all peaks.

[b] We estimate this % purity by using peak abundance from the deconvoluted mass spectrum after LCMS of the entire chromatogram; the percentage represents the maximum abundance of the desired compound mass relative to the abundance of all other peaks within a 4-kDa window, above 1%, of the desired mass. We note that mass spectrometry is only partially quantitative, and purity calculated in this way depends on ionization conditions including cone voltage and temperature. For oligonucleotides containing 2'F-U that were deprotected with aqueous methylamine, we observed impurity peaks (maximum 5-10%) at M-94 and M-52. However, the purity calculations do not include the desired ions when they fly in association with water, acetonitrile, counterions, or buffer. As such, we believe that the numbers in this column underestimate the actual purity.
