# Supplementary material for: Heavily and fully modified RNAs guide efficient SpyCas9-mediated genome editing
Source: Nat Commun. 2018 Jul 6;9:2641. doi: 10.1038/s41467-018-05073-z (PMC6035171; doi:10.1038/s41467-018-05073-z)
Supplement: Supplementary file 3 — Description of Additional Supplementary Files [file 41467_2018_5073_MOESM3_ESM.pdf]

Description of Additional Supplementary Files

File Name: Supplementary Data 1
Description: Raw data for genome editing experiments in HEK293T-TLR cells and hESCs
